# Supplementary material for: SENP6 regulates localization and nuclear condensation of DNA damage response proteins by group deSUMOylation
Source: Nat Commun. 2023 Sep 22;14:5893. doi: 10.1038/s41467-023-41623-w (PMC10514054; doi:10.1038/s41467-023-41623-w)
Supplement: Supplementary file 1 — Supplementary Information [file 41467_2023_41623_MOESM1_ESM.pdf]

SENP6 regulates localization and nuclear condensation of DNA damage response proteins by group deSUMOylation

Laura A. Claessens[1], Matty Verlaan-de Vries[1], Ilona J. de Graaf[1], Alfred C.O. Vertegaal[1*]

1. Cell and Chemical Biology, Leiden University Medical Centre, Leiden, The Netherlands.

* Correspondence: vertegaal@lumc.nl

Supplement

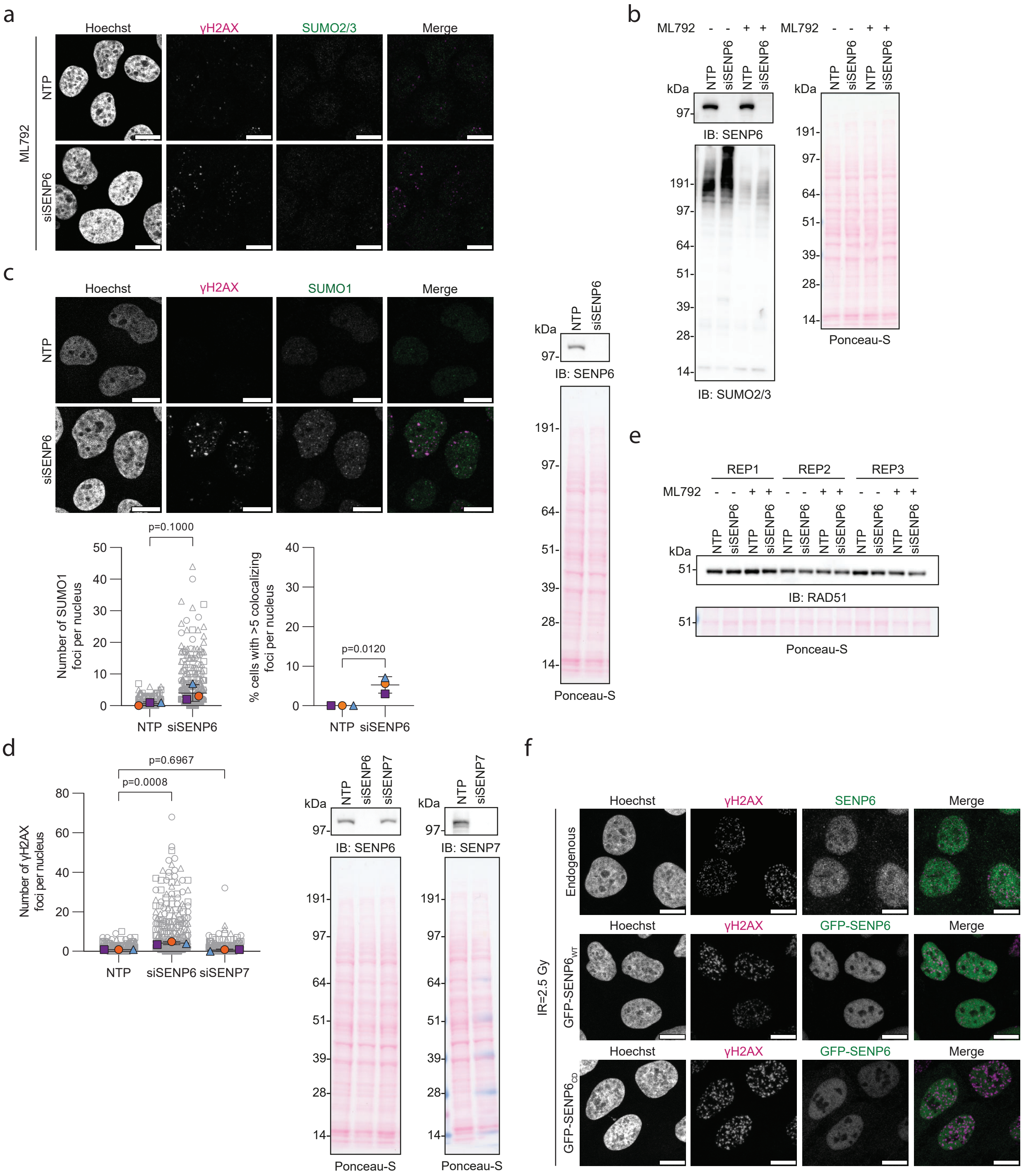
a
Hoechst
γH2AX
SUMO2/3
Merge
ML792
NTP
siSENP6
b
ML792 - - + +
NTP siSENP6 NTP siSENP6
kDa
97-
IB: SENP6
191-
97-
64-
51-
39-
28-
14-
IB: SUMO2/3
Ponceau-S
c
Hoechst
γH2AX
SUMO1
Merge
NTP
siSENP6
p=0.1000
Number of SUMO1 foci per nucleus
NTP siSENP6
p=0.0120
% cells with >5 colocalizing foci per nucleus
NTP siSENP6
IB: SENP6
Ponceau-S
e
REP1 REP2 REP3
ML792
kDa
51-
IB: RAD51
Ponceau-S
d
p=0.6967
p=0.0008
Number of γH2AX foci per nucleus
NTP siSENP6 siSENP7
IB: SENP6
IB: SENP7
Ponceau-S
f
IR=2.5 Gy
Endogenous
GFP-SENP6WT
GFP-SENP6CD
Hoechst
γH2AX
SENP6
GFP-SENP6
Merge

Supplementary Fig 1. DNA damage observed after SENP6 knockdown is induced through excessive SUMOylation. a Representative images corresponding to Fig. 1b, n=3. **b** Lysates from cells corresponding to Fig. 1b-e were immunoblotted for SENP6 and SUMO2/3, n=3. Equal loading was verified by Ponceau-S staining. A representative blot is shown. **c** U2OS were treated with SENP6 (siSENP6) or nontargeting (NTP) siRNAs for 72 h and analyzed by immunofluorescence staining for γH2AX (magenta) and SUMO1 (green), n=3. Left graph: quantification of number of SUMO1 foci per nucleus. Data are shown as pooled cells (from left, n=309,305 cells) (grey), superimposed by the median number of foci per experiment (orange, blue and purple). Right graph: quantification of percentage of cells >5 colocalizing foci per nucleus. Significance was determined with a two-sided unpaired t-test. Cells were also analyzed by immunoblotting for SENP6, n=3. Equal loading was verified by ponceau-S staining. A representative blot is shown. **d** U2OS were treated with SENP6 siRNAs (siSENP6), SENP7 siRNAs (siSENP7) or nontargeting siRNAs (NTP) for 72 h and analyzed by immunofluorescence staining for γH2AX, n=3. Quantification of number of γH2AX foci per nucleus. Data are shown as pooled cells (from left, n=317,327,366 cells) (grey), superimposed by the median number of foci per experiment (orange, blue and purple). Significance was determined by two-sided one-way ANOVA with Dunnett's multiple comparison test. Cells were also analyzed by immunoblotting for SENP6 and SENP7, n=3. Equal loading was verified by ponceau-S staining. Representative blots are shown. **c,d** Error bars in graphs represent mean ±SD. Independent experiments are visualized by unique symbols. **e** Lysates from cells in **(b)** were immunoblotted for RAD51, n=3. Equal loading was verified by Ponceau-S staining. **f** Top panel: U2OS were treated with ionizing radiation (IR) and analyzed by immunofluorescence staining for γH2AX (magenta) and SENP6 (green) after 1 h, n=173 cells across 3 experiments. Middle and bottom panel: U2OS stably expressing wildtype GFP-SENP6 (GFP-$SENP6_{WT}$) and a catalytic dead GFP-SENP6 (GFP-$SENP6_{CD}$) were treated with IR and analyzed by immunofluorescence staining for γH2AX (magenta) and GFP (green) after 1 h, n(GFP-$SENP6_{WT}$)=148 and n(GFP-$SENP6_{CD}$)=121 cells across 3 experiments. Representative images are shown. **a,c,f** Scale bar: 10 µm.

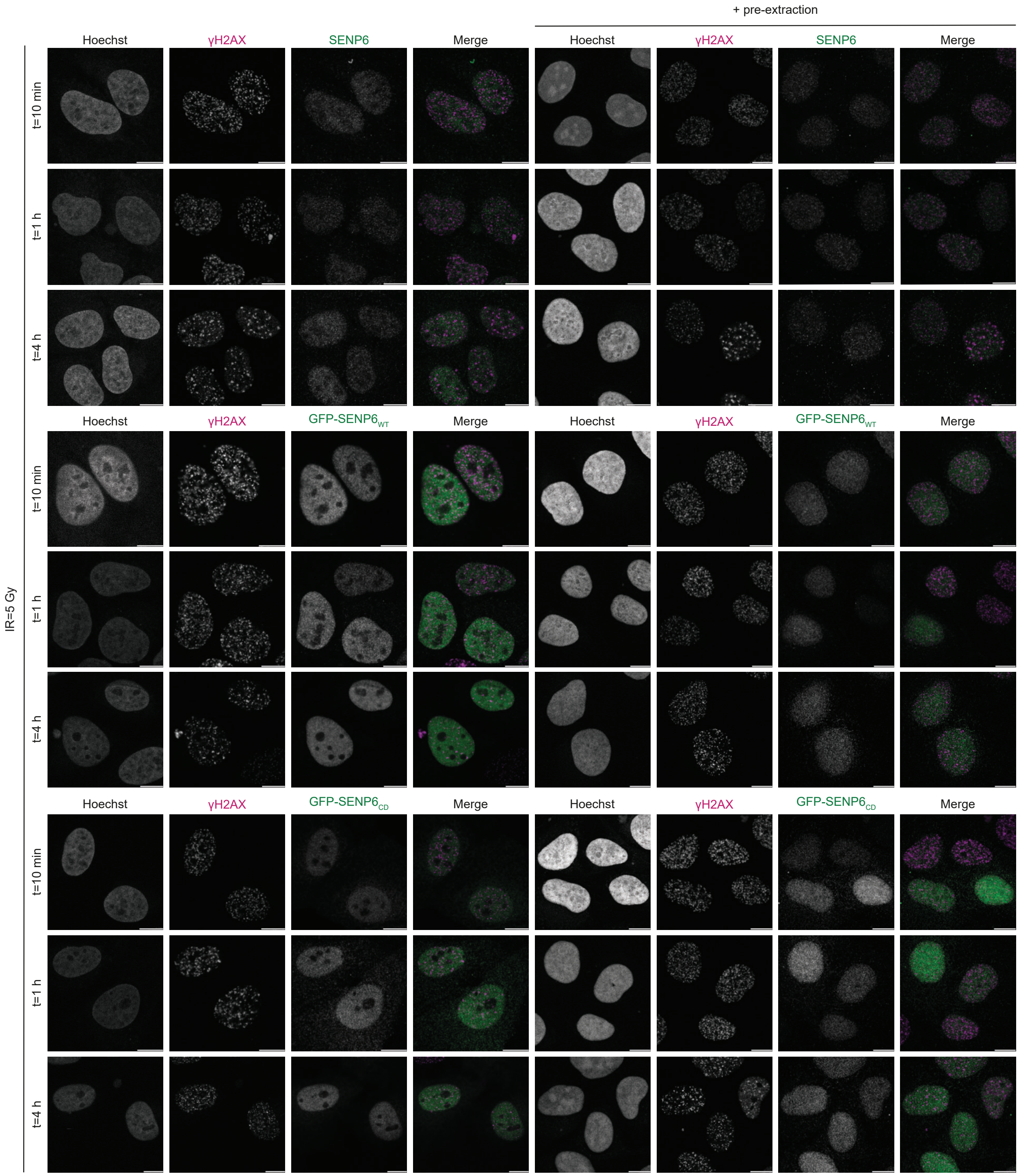
+ pre-extraction
Hoechst
γH2AX
SENP6
Merge
GFP-SENP6WT
GFP-SENP6CD
t=10 min
t=1 h
t=4 h
IR=5 Gy

Supplementary Fig 2. SENP6 does not localize to IR-induced DNA damage foci. Top left panel: U2OS were treated with ionizing radiation (IR) and analyzed by immunofluorescence staining for γH2AX (magenta) and SENP6 (green) at the indicated timepoints. n(10 min)=98, n(1 h)=81, n(4 h)=95 cells. Middle and bottom left panel: U2OS cells stably expressing wildtype GFP-SENP6 (GFP-$SENP6_{WT}$) and a catalytic dead GFP-SENP6 (GFP-$SENP6_{CD}$) were treated with IR and analyzed by immunofluorescence staining for γH2AX (magenta) and GFP (green) at the indicated timepoints. n(10 min)=71, n(1 h)=82, n(4 h)=68 cells and n(10 min)=56, n(1 h)=48, n(4 h)=50 cells, respectively. Right panel shows the same experimental set-up as in the left panel, but a pre-extraction step was included before fixing the cells for immunofluorescence staining. n(10 min)=100, n(1 h)=104, n(4 h)=81 cells; n(10 min)=59, n(1 h)=70, n(4 h)=42 cells and n(10 min)=46, n(1 h)=48, n(4 h)=56 cells, respectively. Representative images are shown. Scale bar: 10 µm.

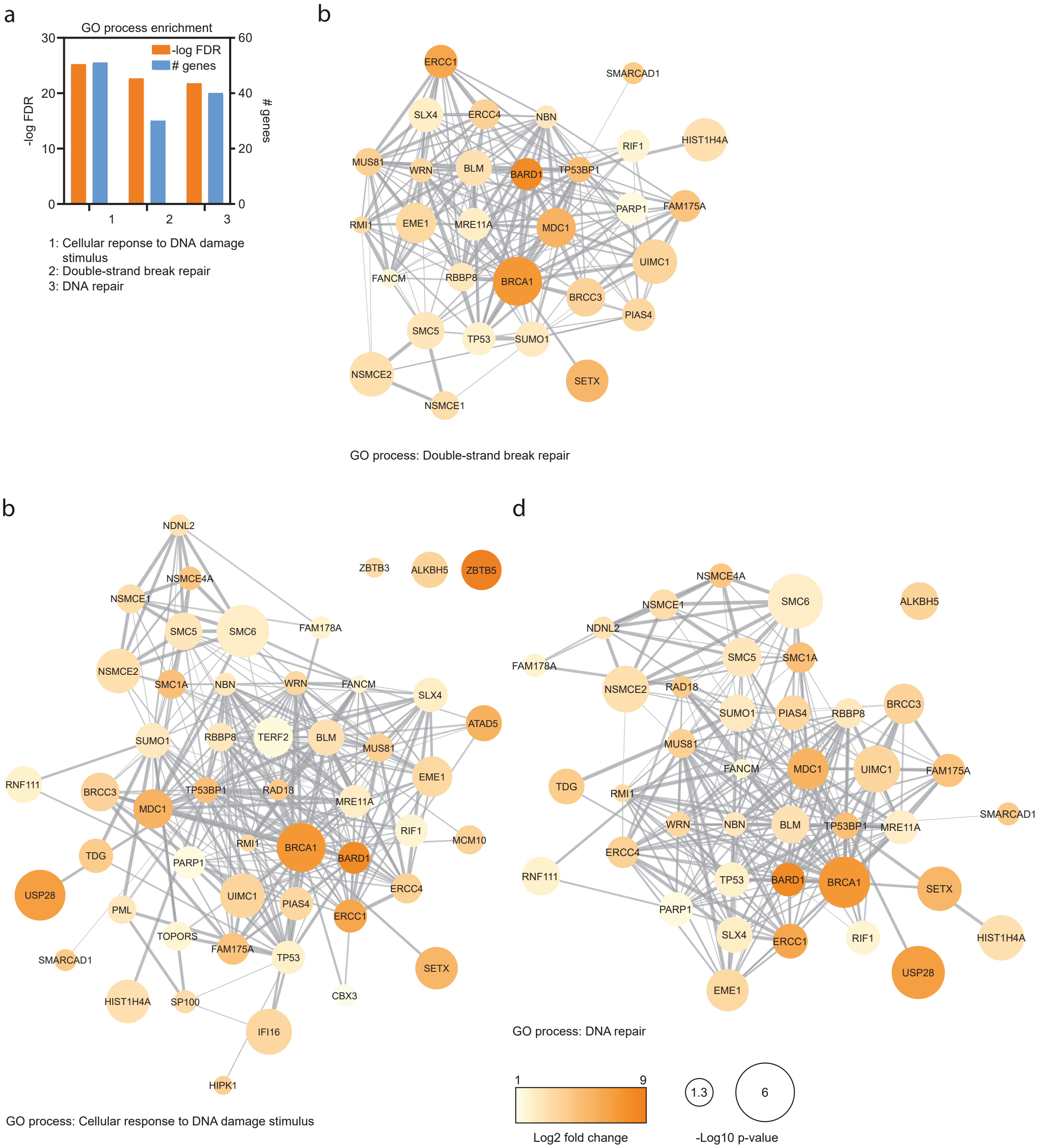

a
GO process enrichment
-log FDR
genes
30
20
10
0
60
40
20
0
1
2
3
1: Cellular reponse to DNA damage stimulus
2: Double-strand break repair
3: DNA repair
b
ERCC1
SMARCAD1
SLX4
ERCC4
NBN
RIF1
HIST1H4A
MUS81
WRN
BLM
BARD1
TP53BP1
PARP1
FAM175A
RMI1
EME1
MRE11A
MDC1
UIMC1
FANCM
RBBP8
BRCA1
BRCC3
PIAS4
SMC5
TP53
SUMO1
NSMCE2
SETX
NSMCE1
GO process: Double-strand break repair
b
NDNL2
ZBTB3
ALKBH5
ZBTB5
NSMCE4A
NSMCE1
SMC5
SMC6
FAM178A
NSMCE2
SMC1A
NBN
WRN
FANCM
SLX4
ATAD5
SUMO1
RBBP8
TERF2
BLM
MUS81
RNF111
BRCC3
TP53BP1
RAD18
EME1
MDC1
MRE11A
RIF1
MCM10
TDG
RMI1
BRCA1
BARD1
PARP1
USP28
UIMC1
PIAS4
ERCC4
PML
ERCC1
TOPORS
SMARCAD1
FAM175A
TP53
SETX
CBX3
HIST1H4A
SP100
IFI16
HIPK1
GO process: Cellular response to DNA damage stimulus
d
NSMCE4A
SMC6
ALKBH5
NSMCE1
NDNL2
SMC5
SMC1A
FAM178A
NSMCE2
RAD18
BRCC3
SUMO1
PIAS4
RBBP8
MUS81
FANCM
MDC1
UIMC1
FAM175A
TDG
RMI1
SMARCAD1
WRN
NBN
BLM
TP53BP1
MRE11A
ERCC4
RNF111
TP53
BARD1
BRCA1
SETX
PARP1
HIST1H4A
SLX4
ERCC1
RIF1
USP28
EME1
GO process: DNA repair
1
9
Log2 fold change
1.3
6
-Log10 p-value

Supplementary Fig 3. Identification of SENP6 targets by mass-spectrometry. a Gene ontology (GO) enrichment analysis on the 180 SENP6 targets showing a significant increase in SUMO2 conjugation after SENP6 knockdown (Source proteomics data: ProteomeXchange Consortium via the PRIDE partner repository PXD011963). The enrichment (-log FDR) and the number of genes (# genes) of the top GO processes involving the DNA damage response are plotted. **b-d** Protein STRING networks of the top GO processes involving the DNA damage response shown in **(a)**. The size of the nodes represents the -log10 p-value and the color of the nodes the log2 fold change of the enriched proteins after SENP6 knockdown compared with the nontargeting control. Size and color grading is indicated by the legend included in **(d)**. The width of the edges connecting the nodes represents the string score for protein-protein interactions curated from experimental data.

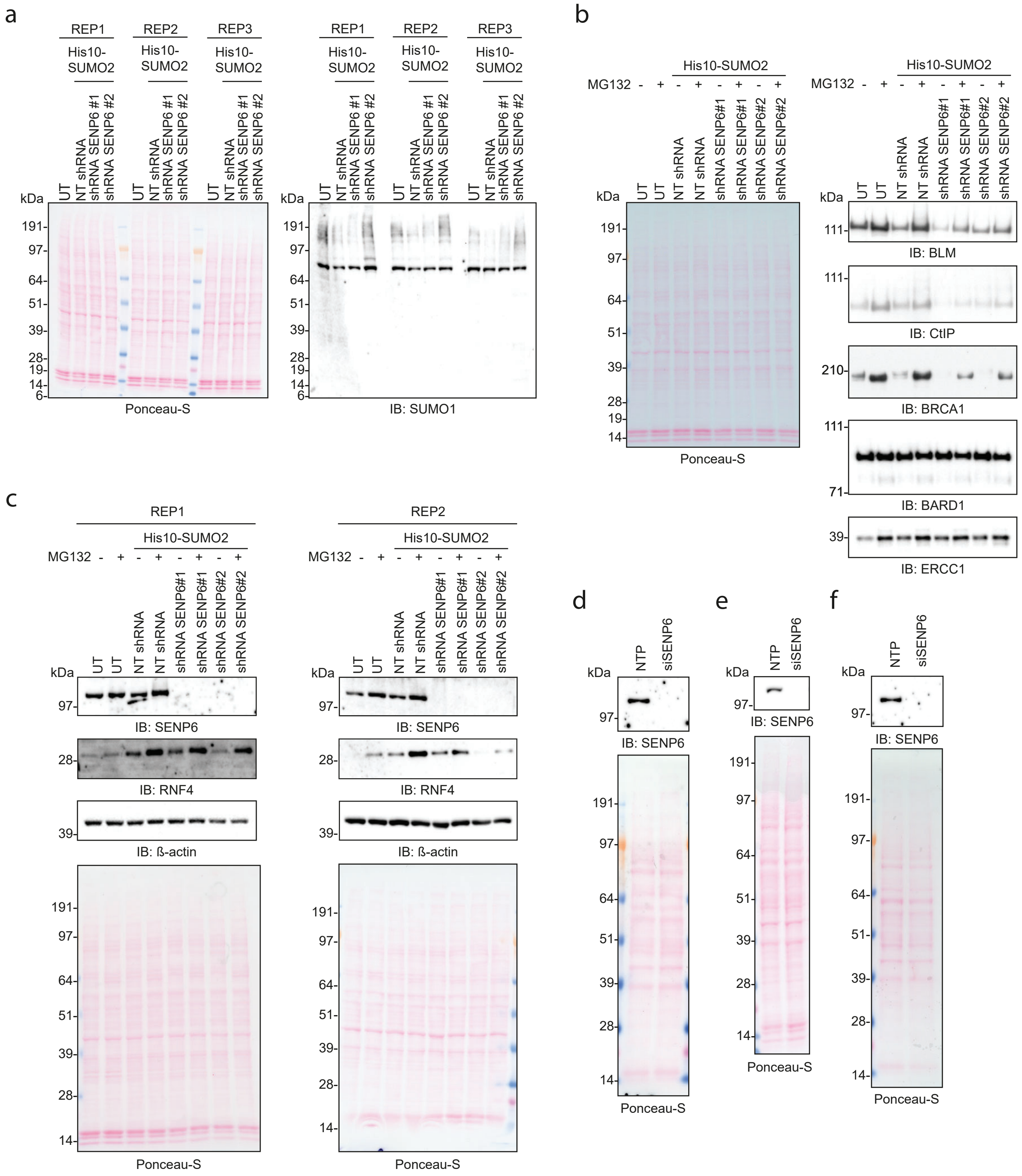

a
REP1
REP2
REP3
His10-SUMO2
UT
NT shRNA
shRNA SENP6 #1
shRNA SENP6 #2
kDa
191-
97-
64-
51-
39-
28-
19-
14-
6-
Ponceau-S
IB: SUMO1
b
His10-SUMO2
MG132
UT
NT shRNA
shRNA SENP6#1
shRNA SENP6#2
Ponceau-S
IB: BLM
IB: CtIP
IB: BRCA1
IB: BARD1
IB: ERCC1
111-
210-
71-
39-
c
REP1
REP2
His10-SUMO2
MG132
IB: SENP6
IB: RNF4
IB: ß-actin
Ponceau-S
d
e
f
NTP
siSENP6
IB: SENP6
Ponceau-S

Supplementary Fig 4. Potential role for SENP6 in regulation of DDR protein turnover. a U2OS stably expressing His10-SUMO2 were infected with lentiviruses encoding shRNAs against SENP6 (shRNA SENP6#1 and #2) or a nontargeting control shRNA (NT shRNA). Cells were lysed three days post infection and analyzed by immunoblotting for SUMO1, n=3. Equal loading was verified by Ponceau-S staining. **b** U2OS stably expressing His10-SUMO2 were infected with lentiviruses encoding shRNAs against SENP6 (shRNA SENP6#1 and #2) or a nontargeting control shRNA (NT shRNA) and lysed three days post infection. Where indicated, cells were treated with 10 µM MG132 for 6-8 h prior to lysis. Total lysates were immunoblotted for the indicated DDR proteins, n=2. Equal loading was verified by Ponceau-S staining. Representative blots are shown. **c** Lysates in **(b)** were also analyzed by immunoblotting for SENP6 and RNF4, n=2. Equal loading was verified by ß-actin and Ponceau-S staining. **d** Immunoblot for SENP6, corresponding to Fig. 6a. Equal loading was verified by Ponceau-S staining. **e** Immunoblot for SENP6, corresponding to Fig. 6b. Equal loading was verified by Ponceau-S staining. **f** Immunoblot for SENP6, corresponding to Fig. 6c-h. Equal loading was verified by Ponceau-S staining.

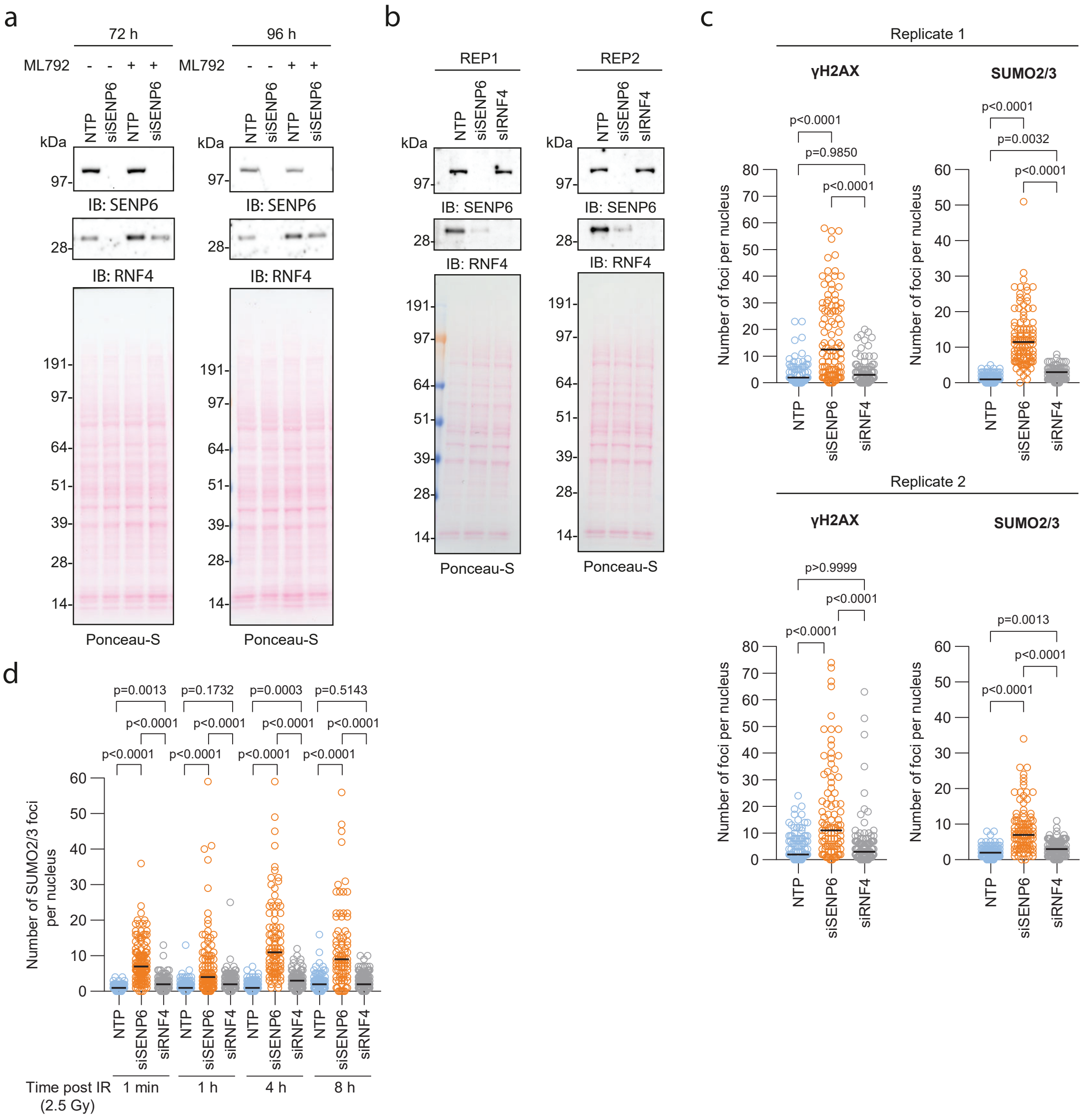

a
72 h
96 h
ML792 - - + +
NTP
siSENP6
kDa
97-
IB: SENP6
28-
IB: RNF4
191-
64-
51-
39-
14-
Ponceau-S
b
REP1
REP2
siRNF4
c
Replicate 1
γH2AX
SUMO2/3
p<0.0001
p=0.9850
p=0.0032
Number of foci per nucleus
Replicate 2
p>0.9999
p=0.0013
d
p=0.1732
p=0.0003
p=0.5143
Number of SUMO2/3 foci per nucleus
Time post IR (2.5 Gy)
1 min
1 h
4 h
8 h

Supplementary Fig 5. Genomic instability after SENP6 depletion is not indirectly caused by RNF4 depletion. **a** Lysates from cells corresponding to Fig. 1b-e and supplementary Fig. 1a-b (left panel) and Fig. 7c-d (right panel) were immunoblotted for SENP6 and RNF4, n=3. Equal loading was verified by Ponceau-S staining. Representative blots are shown. **b** U2OS were treated with SENP6 siRNAs (siSENP6), RNF4 siRNA (siRNF4) or nontargeting siRNAs (NTP) for 72 h and analyzed by immunoblotting for SENP6 and RNF4, n=2. Equal loading was verified by Ponceau-S staining. **c** NTP, siSENP6 and siRNF4 cells in **b** were analyzed by immunofluorescence staining for γH2AX and SUMO2/3, n=2. Number of γH2AX and SUMO2/3 foci per nucleus were quantified. Each datapoint represents a single cell (from left, n(1)=86,100,84 and n(2)=105,93,106 cells). Lines represent median. **d** U2OS were treated with siSENP6, siRNF4 or NTP siRNA for 72 h, treated with ionizing radiation (IR) and analyzed by immunofluorescence staining for γH2AX and SUMO2/3 at the indicated timepoints. Number of SUMO2/3 foci per nucleus was quantified. Each datapoint represents a single cell (from left, n=103,77,103,82,119,95,88,80,116,103,96,107 cells. Lines represent the median. Significance was determined by two-sided one-way ANOVA with Dunnett's multiple comparison test at each timepoint.

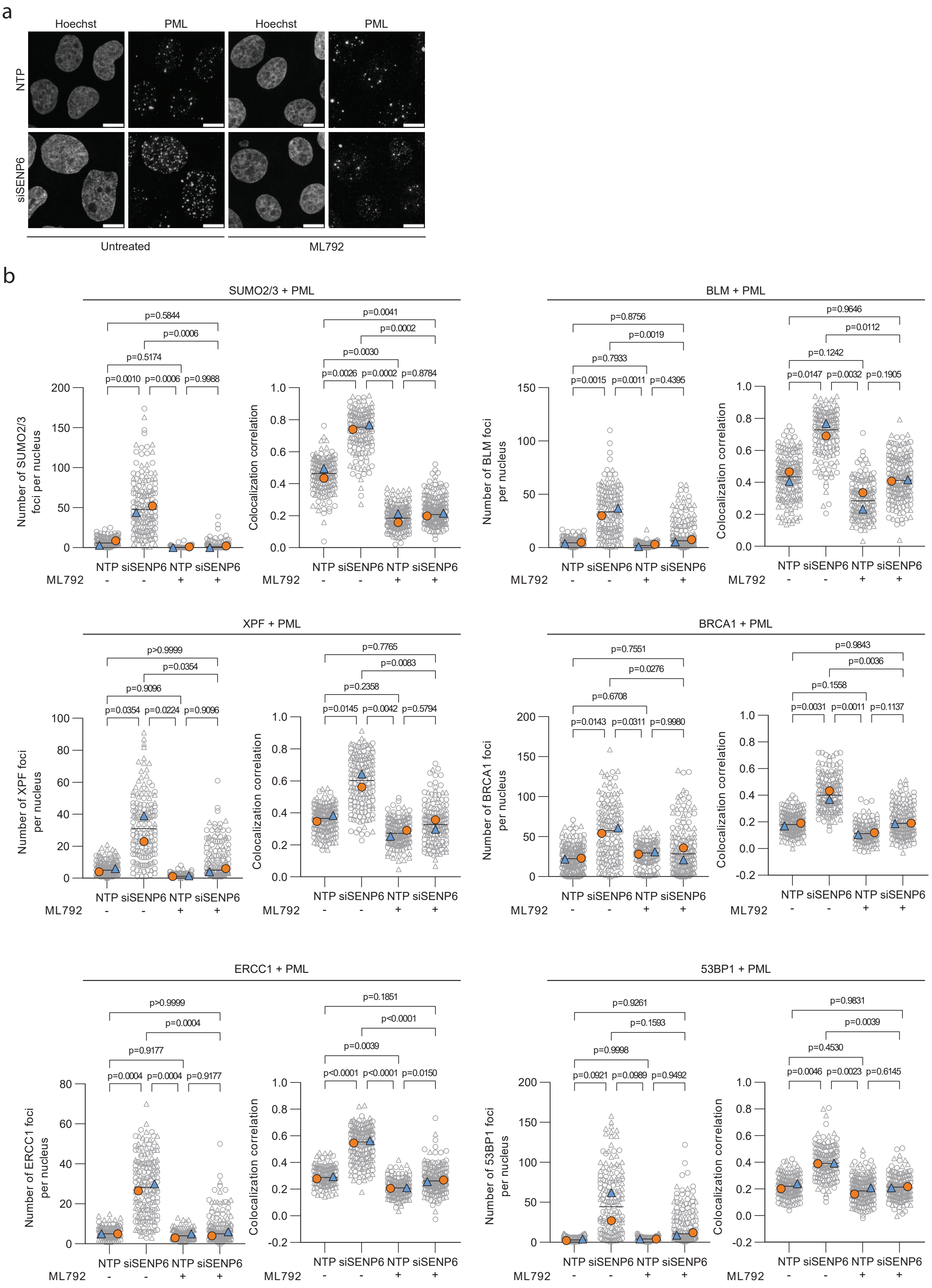

a
Hoechst
PML
Hoechst
PML
NTP
siSENP6
Untreated
ML792
b
SUMO2/3 + PML
p=0.5844
p=0.0006
p=0.5174
p=0.0010 p=0.0006 p=0.9988
Number of SUMO2/3 foci per nucleus
p=0.0041
p=0.0002
p=0.0030
p=0.0026 p=0.0002 p=0.8784
Colocalization correlation
NTP siSENP6 NTP siSENP6
ML792 - - + +
BLM + PML
p=0.8756
p=0.0019
p=0.7933
p=0.0015 p=0.0011 p=0.4395
Number of BLM foci per nucleus
p=0.9646
p=0.0112
p=0.1242
p=0.0147 p=0.0032 p=0.1905
Colocalization correlation
XPF + PML
p>0.9999
p=0.0354
p=0.9096
p=0.0354 p=0.0224 p=0.9096
Number of XPF foci per nucleus
p=0.7765
p=0.0083
p=0.2358
p=0.0145 p=0.0042 p=0.5794
Colocalization correlation
BRCA1 + PML
p=0.7551
p=0.0276
p=0.6708
p=0.0143 p=0.0311 p=0.9980
Number of BRCA1 foci per nucleus
p=0.9843
p=0.0036
p=0.1558
p=0.0031 p=0.0011 p=0.1137
Colocalization correlation
ERCC1 + PML
p>0.9999
p=0.0004
p=0.9177
p=0.0004 p=0.0004 p=0.9177
Number of ERCC1 foci per nucleus
p=0.1851
p<0.0001
p=0.0039
p<0.0001 p<0.0001 p=0.0150
Colocalization correlation
53BP1 + PML
p=0.9261
p=0.1593
p=0.9998
p=0.0921 p=0.0989 p=0.9492
Number of 53BP1 foci per nucleus
p=0.9831
p=0.0039
p=0.4530
p=0.0046 p=0.0023 p=0.6145
Colocalization correlation

Supplementary Fig 6. PolySUMOylated DDR proteins accumulate and localize to PML bodies. a Microscopy images corresponding to Fig. 7c. U2OS were treated with SENP6 siRNAS (siSENP6) or nontargeting siRNAs (NTP) for 96 h and treated with or without 1 µM ML792 for 24 h, n=6. Cells were analyzed by immunofluorescence staining for PML. Representative images are shown. Scale bar: 10 µm. **b** Quantification corresponding to Fig. 7d for SUMO2/3 or DDR foci and PML bodies. The left graph shows the number of SUMO2/3 or DDR foci per nucleus. In the right graph colocalization of SUMO2/3 or DDR protein with PML bodies is estimated by Pearson correlation. Data are shown as pooled cells (grey), superimposed by the median percentage or correlation per experiment (blue and orange). Independent experiments are visualized by unique symbols. Lines represent the mean. Significance was determined by two-sided one-way ANOVA with Tukey's multiple comparison test.

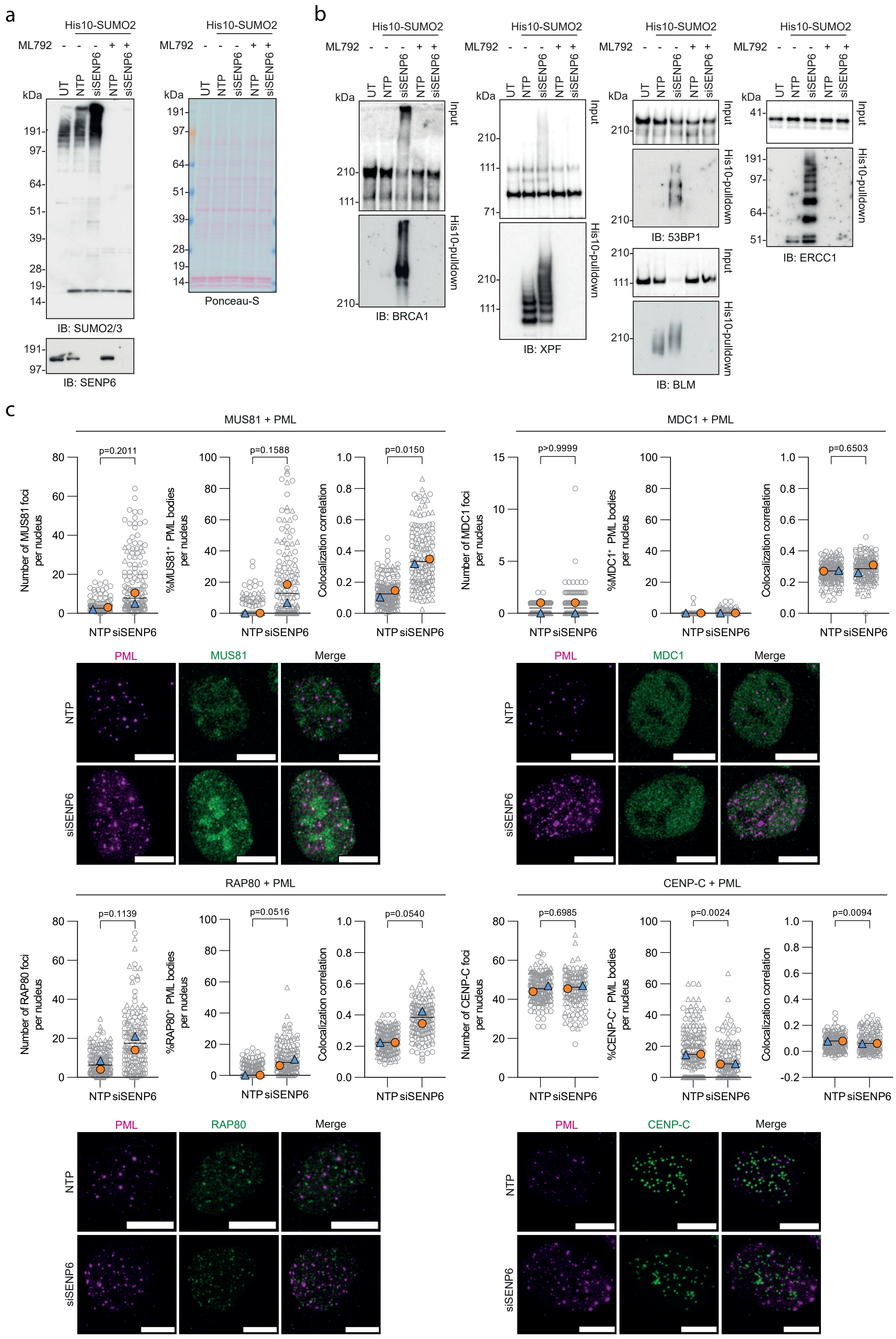

a
His10-SUMO2
ML792
UT
NTP
siSENP6
kDa
191
97
64
51
39
28
19
14
IB: SUMO2/3
IB: SENP6
Ponceau-S
b
Input
His10-pulldown
IB: BRCA1
IB: XPF
IB: 53BP1
IB: BLM
IB: ERCC1
210
111
71
41
c
MUS81 + PML
MDC1 + PML
RAP80 + PML
CENP-C + PML
Number of MUS81 foci per nucleus
%MUS81⁺ PML bodies per nucleus
Colocalization correlation
Number of MDC1 foci per nucleus
%MDC1⁺ PML bodies per nucleus
Number of RAP80 foci per nucleus
%RAP80⁺ PML bodies per nucleus
Number of CENP-C foci per nucleus
%CENP-C⁺ PML bodies per nucleus
p=0.2011
p=0.1588
p=0.0150
p>0.9999
p=0.6503
p=0.1139
p=0.0516
p=0.0540
p=0.6985
p=0.0024
p=0.0094
NTP
siSENP6
PML
MUS81
MDC1
RAP80
CENP-C
Merge

Supplementary Fig 7. SUMO2/3 polymerization of proteins is required but not sufficient for PML body localization. Total lysates corresponding to Fig. 7b were also immunoblotted for SENP6 and SUMO2/3 **(a)** and the indicated DDR proteins **(b)**, n=2. Same loading was used and verified by Ponceau-S staining. Representative blots are shown. **c** Cells corresponding to Fig. 7c-d were also stained for PML (magenta), and MUS81, RAP80, MDC1 or CENP-C (green). Left graph: number of DDR foci per nucleus; middle graph: colocalization quantified as percentage of PML bodies that colocalize with a DDR protein per nucleus; right graph: colocalization estimated by Pearson correlation. Data are shown as pooled cells (MUS81 from left, n=208,147; RAP80 from left, n=184,162; MDC1 from left, n=185,166; CENP-C from left, n=195,141 cells) (grey), superimposed by the median per experiment (blue and orange). Independent experiments are visualized by unique symbols. Lines represent the mean. Significance was determined with a two-sided unpaired t-test. For microscopy inserts, representative images of cells are shown. Scale bar: 10 µm.

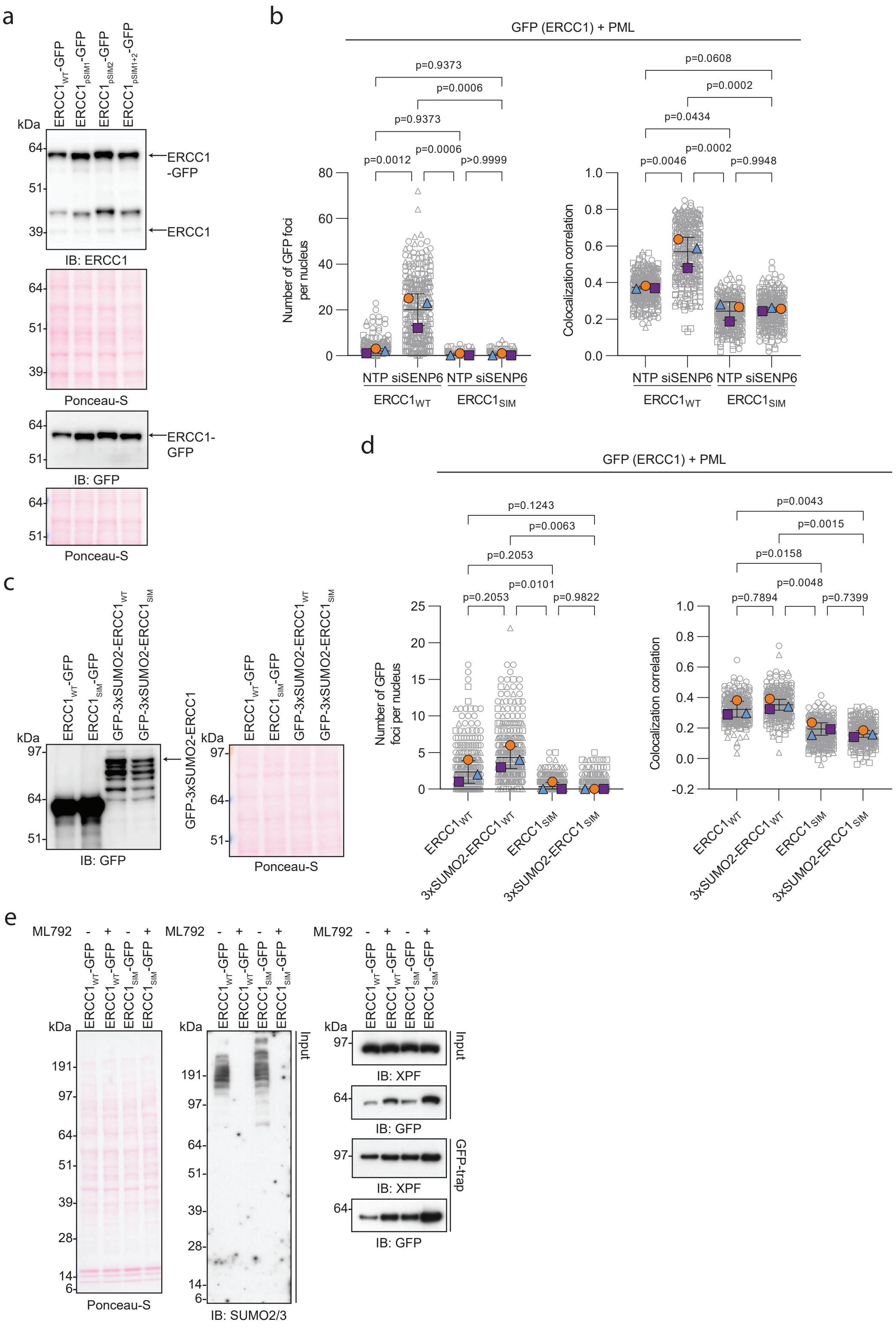
a
ERCC1WT-GFP
ERCC1pSIM1-GFP
ERCC1pSIM2-GFP
ERCC1pSIM1+2-GFP
kDa
64
51
39
ERCC1 -GFP
ERCC1
IB: ERCC1
Ponceau-S
IB: GFP
ERCC1- GFP
Ponceau-S
b
GFP (ERCC1) + PML
p=0.9373
p=0.0006
p=0.9373
p=0.0006
p=0.0012
p>0.9999
Number of GFP foci per nucleus
NTP siSENP6 NTP siSENP6
ERCC1WT ERCC1SIM
p=0.0608
p=0.0002
p=0.0434
p=0.0002
p=0.0046
p=0.9948
Colocalization correlation
c
ERCC1WT-GFP
ERCC1SIM-GFP
GFP-3xSUMO2-ERCC1WT
GFP-3xSUMO2-ERCC1SIM
kDa
97
64
51
GFP-3xSUMO2-ERCC1
IB: GFP
Ponceau-S
d
GFP (ERCC1) + PML
p=0.1243
p=0.0063
p=0.2053
p=0.0101
p=0.2053
p=0.9822
Number of GFP foci per nucleus
ERCC1WT
3xSUMO2-ERCC1WT
ERCC1SIM
3xSUMO2-ERCC1SIM
p=0.0043
p=0.0015
p=0.0158
p=0.0048
p=0.7894
p=0.7399
Colocalization correlation
e
ML792 - + - +
ERCC1WT-GFP
ERCC1WT-GFP
ERCC1SIM-GFP
ERCC1SIM-GFP
kDa
191
97
64
51
39
28
14
6
Ponceau-S
Input
IB: SUMO2/3
IB: XPF
IB: GFP
GFP-trap
IB: XPF
IB: GFP

Supplementary Fig 8. A functional SIM domain is required for ERCC1 accumulation and localization to PML bodies. a U2OS stably expressing ERCC1-GFP or ERCC1-GFP with pSIM1, pSIM2 or both mutated were analyzed by immunoblotting for ERCC1 and GFP, n=3. Equal loading was confirmed with Ponceau-S staining. Representative blots are shown. **b** Quantification corresponding to Fig. 8d. The left graph shows the number of GFP foci per nucleus. In the right graph colocalization of GFP with PML bodies is estimated by Pearson correlation. **c** U2OS stably expressing GFP-3xSUMO2-$ERCC1_{WT}$ and GFP-3xSUMO2-$ERCC1_{SIM,}$ were immunoblotted for GFP, n=1. Equal loading was confirmed with Ponceau-S staining. **d** Quantification corresponding to Fig. 8e. The left graph shows the number of GFP foci per nucleus. In the right graph colocalization of GFP with PML bodies is estimated by Pearson correlation. **b,d** Data are shown as pooled cells (grey), superimposed by the median per experiment (blue, orange and purple). Independent experiments are visualized by unique symbols. Error bars represent mean ±SD. Significance was determined by two-sided one-way ANOVA with Tukey's multiple comparison test. **e** Western blot images corresponding to Fig. 8f. U2OS stably expressing ERCC1-GFP wildtype ($ERCC1_{WT}$-GFP) or ERCC1-GFP SIM mutant ($ERCC1_{SIM}$-GFP) were treated with or without 1 µM ML792 for 24 h. $ERCC1_{WT/SIM}$-GFP were purified and samples were analyzed by immunoblotting for GFP and XPF. Equal loading of lysates was verified by Ponceau-S staining. Representative blots are shown.

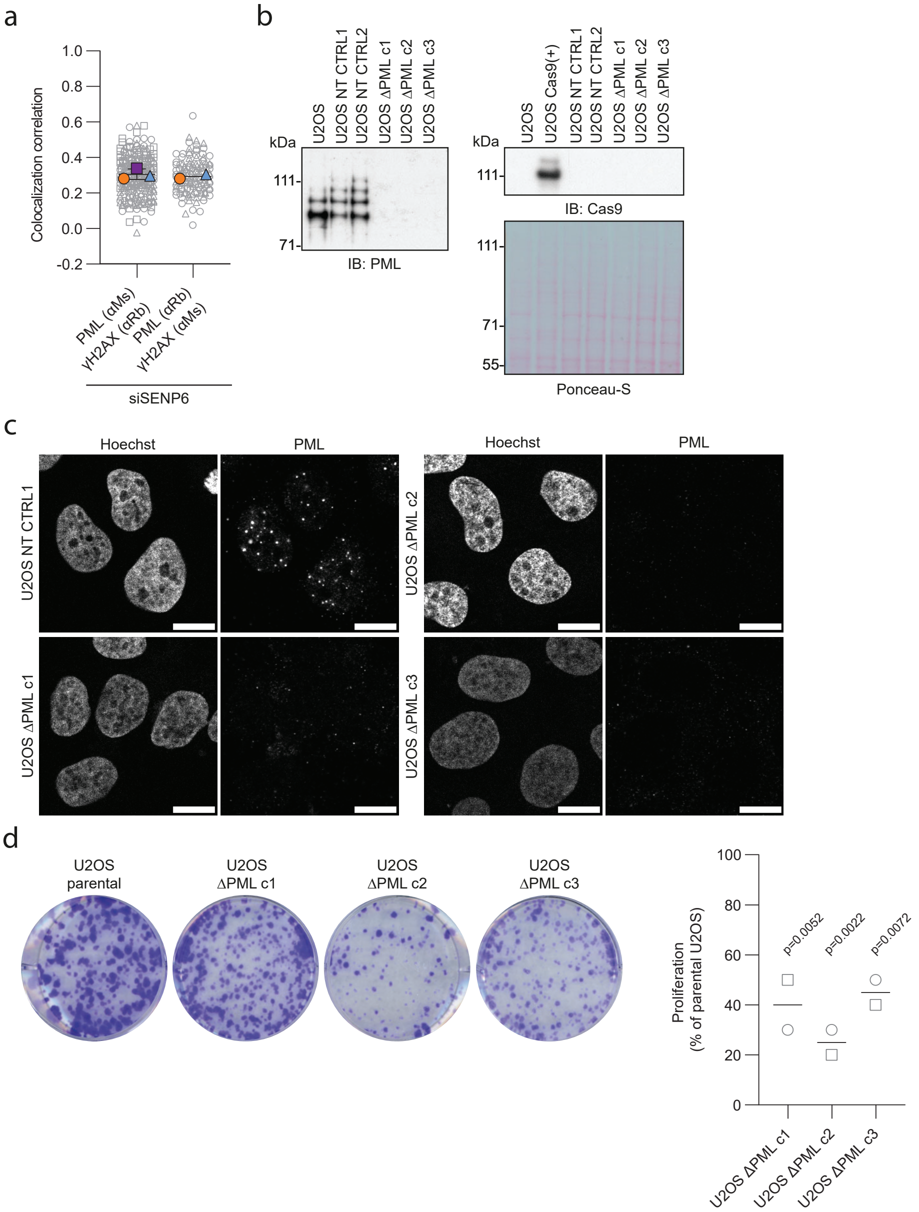

a
Colocalization correlation
1.0
0.8
0.6
0.4
0.2
0.0
-0.2
PML (αMs) γH2AX (αRb)
PML (αRb) γH2AX (αMs)
siSENP6
b
kDa
U2OS
U2OS NT CTRL1
U2OS NT CTRL2
U2OS ΔPML c1
U2OS ΔPML c2
U2OS ΔPML c3
111-
71-
IB: PML
U2OS Cas9(+)
IB: Cas9
55-
Ponceau-S
c
Hoechst
PML
U2OS NT CTRL1
U2OS ΔPML c1
U2OS ΔPML c2
U2OS ΔPML c3
d
U2OS parental
U2OS ΔPML c1
U2OS ΔPML c2
U2OS ΔPML c3
Proliferation (% of parental U2OS)
100
80
60
40
20
0
p=0.0052
p=0.0022
p=0.0072

Supplementary Fig 9. PML is not causally involved in the genomic instability induced by SENP6 knockdown. a Quantification corresponding to Fig. 9a. γH2AX and PML colocalization was estimated by Pearson correlation. Data are shown as pooled cells (from left, n=232,125 cells) (grey), superimposed by the median correlation per experiment (blue, orange and purple). Data is shown for siSENP6-treated cells from n=3 for α-PML mouse (Ms) and α-γH2AX rabbit (Rb), and n=2 for α-PML Rb and α-γH2AX Ms costaining. Error bars represent mean ±SD. **b** Cell lysates from U2OS and U2OS ΔPML cells (c1, c2, c3) were immunoblotted for PML and Cas9 to confirm knockout of PML and no stable integration of GFP-Cas9. U2OS transiently transfected with the GFP-Cas9-expressing plasmid were included as positive control for Cas9 immunoblotting. Two clones from U2OS transfected with a non-targeting gRNA (NT CTRL1 and NT CTRL2) are shown as additional control cell lines. Equal loading was verified by Ponceau-S staining. **c** U2OS ΔPML cells (c1, c2, c3) were analyzed by immunofluorescence staining for PML. A clone from U2OS transfected with a non-targeting gRNA (NT CTRL1) is shown as additional control cell line. Representative images are shown. Scale bar: 10 µm. **d** Colony forming assay of U2OS and U2OS ΔPML cells. Cells were seeded at the same density and fixed and stained with crystal violet after 14 days, n=2. Representative images (left) and quantification of solubilized crystal violet (right) are shown. Independent experiments are visualized by unique symbols. Cell proliferation is shown as percentage, with parental U2OS set at 100% proliferation. Significance between U2OS ΔPML cells and parental U2OS was determined by two-sided one-way ANOVA with Dunnett's multiple comparison test.

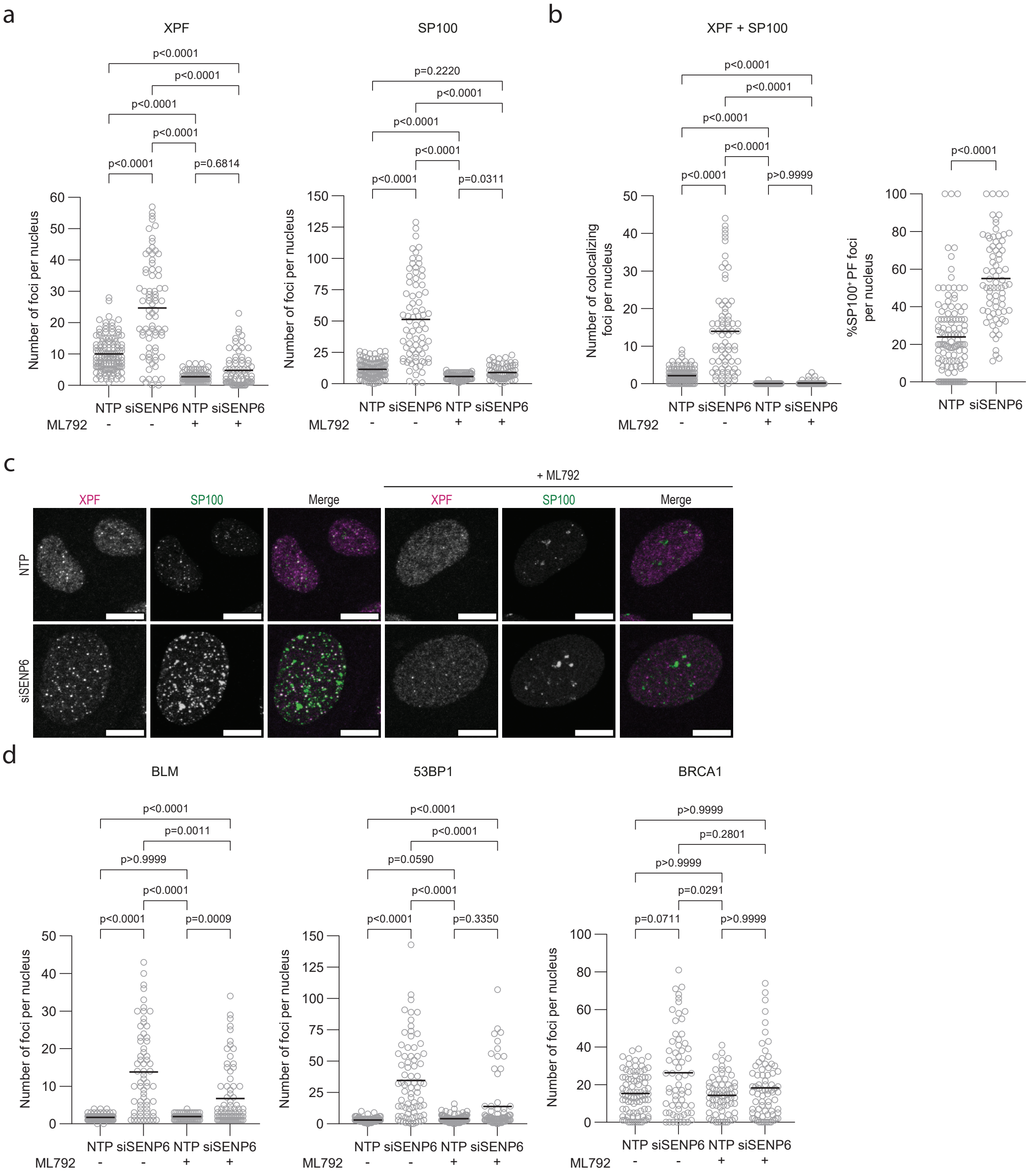
a
XPF
SP100
b
XPF + SP100
p<0.0001
p=0.6814
p=0.2220
p=0.0311
p>0.9999
Number of foci per nucleus
Number of colocalizing foci per nucleus
%SP100+ PF foci per nucleus
NTP siSENP6 NTP siSENP6
ML792 - - + +
c
XPF
SP100
Merge
+ ML792
NTP
siSENP6
d
BLM
53BP1
BRCA1
p=0.0011
p>0.9999
p=0.0009
p=0.0590
p=0.3350
p=0.2801
p=0.0291
p=0.0711

Supplementary Fig 10. Accumulation of DDR proteins in condensates after SENP6 depletion can occur independently of PML. a U2OS were treated with SENP6 siRNAs (siSENP6) or nontargeting siRNAs (NTP) for 96 h and treated with or without 1 µM ML792 for 24 h. Cells were analyzed by immunofluorescence staining for XPF (magenta) and SP100 (green). Number of XPF (left panel) and SP100 (right panel) foci per nucleus was quantified. Significance was determined by two-sided one-way ANOVA with Dunnett's multiple comparison test. **b** Number of XPF and SP100 colocalizing foci per nucleus was quantified (left panel). Significance was determined by two-sided one-way ANOVA with Dunnett's multiple comparison test. Percentage of $SP100^{+}$ XPF foci per nucleus was quantified (right panel). Significance was determined with a two-sided Mann-Whitney U test. **a,b** From left, n=121,76,78,73 cells. **c** Representative microscopy images corresponding to **(a-b)**. Scale bar: 10 µM. **d** Cells in **(a-c)** were also stained for BLM (left panel), 53BP1 (middle panel) and BRCA1 (right panel). Number of foci per nucleus was quantified. From left, n(BLM)=92,66,89,78; n(53BP1)=95,76,96,75; n(BRCA1)=89,65,72,76 cells. Significance was determined by one-way ANOVA with Dunnett's multiple comparison test. **a,b,d** Lines represent the median.

Supplementary Table 1. Key resources

Reagent or resource	Source	Identifier
**Antibodies**		
anti-SENP6 (IB[a] 1:100; IF[b] 1:50)	Sigma-Aldrich	WHP0026054M1
anti-SENP7 (IB 1:1000)	Bethyl	A302-995A
anti-SUMO2/3 8A2 (IB 1:250)	University of Iowa	8A2
anti-SUMO2/3 2277 (IF 1:100)	Eurogentec	[1]
anti-SUMO1 2C7 (IB 1:1000; IF 1:100)	Zymed laboratories Inc.	18-2306
anti-PML (IB 1:100; IF 1:500)	Bethyl	A301-167A
anti-PML (IB 1:1000; IF 1:1000)	MBL	M041-3
anti-γH2AX (IB 1:500; IF 1:500)	Merck Millipore	05-636
anti-γH2AX (IB: 1:500)	Cell signaling technology	9718
anti-RNF4 (IB 1:3000)	Eurogentec	[2]
anti-ERCC1 (IF 1:100)	Abcam	ab129267
anti-ERCC1 (IB 1:500-1:1000)	Cell signaling technology	12345
anti-BLM (IB 1:1000; IF 1:200)	Abcam	ab2179
anti-XPF (IB 1:500)	Thermo Fisher Scientific	MS-1381
anti-XPF (IF 1:100)	Santa Cruz	sc-136153
anti-MUS81 (IB 1:500-1:1000; IF 1:250)	ImmuQuest	IQ285
anti-53BP1 (IB 1:1000; IF 1:500)	Bethyl	A300-272A
anti-BRCA1 (IB: 1:500)	Cell signaling technology	9010
anti-BRCA1 (IF 1:1000)	Merck Millipore	07-434
anti-RAP80 (IB 1:1000; IF 1:200)	Bethyl	A300-763A
anti-MDC1 (IB 1:1000; IF 1:500)	Bethyl	A300-052A
anti-CtIP (IB 1:1000; IF 1:200)	Bethyl	A300-488A
anti-EME1 (IB 1:1000)	ImmuQuest	IQ284
anti-BARD1 (IB 1:1000)	Bethyl	A300-263A
anti-Sp100 (IB 1:1000)	Chemicon international	AB1380
anti-RAD51 (IB 1:500)	Cell signaling technology	8875
anti-Ubiquitinated proteins FK2 (IB 1:1000)	Merck Millipore	04-263
anti-CENP-C (IF 1:100)	Merck Millipore	MABE1114
anti-GFP (IB 1:5000; IF 1:500)	Novus Biololgicals	NB600-308
anti-Cas9 (IB 1:1000)	Cell Signaling technology	14697
Alexa-Fluor 568 goat-anti-rabbit / goat-anti-mouse (IF 1:250)	Invitrogen	A11004 / A11011
Alexa-Fluor 488 goat-anti-rabbit / goat-anti-mouse (IF 1:250)	Invitrogen	A11034 / A11001
**Oligonucleotides**		
SMART pool ON-TARGETplus SENP6 siRNA	Dharmacon	L-006044-00-00
ON-TARGETplus RNF4 siRNA	Dharmacon	J-006557-08
SMART pool ON-TARGET siRNA nontargeting	Dharmacon	D-001810-10-
shRNA SENP6 #1-GACAGAACTAACAGAAGAGAA	Sigma-Aldrich MISSION® shRNA library	TRC -004103
shRNA SENP6#2-CACAGGATTAACAACCAAGAA	Sigma-Aldrich MISSION® shRNA library	TRC-004104
Non-targeting shRNA	Sigma-Aldrich MISSION® shRNA library	TRC-SHC002
shRNA RNF4 #1-CATACTCCCAGAAACGCCAGG	Sigma-Aldrich MISSION® shRNA library	TRC-272668

shRNA RNF4 #2- CATACTCCCAGAAACGCCAGG	Sigma-Aldrich MISSION® shRNA library	TRC-284821
ERCC1 pSIM1 FWD: 5'- GCCAGCAAGGAAGAAATTTGCGGCACCCGCCG ACGAGGATGAGGTCCCTC-3'	This study, to generate $ERCC1_{pSIM1}$	N/A
ERCC1 pSIM1 REV: 5'- GAGGGACCTCATCCTCGTCGGCGGGTGCCGCAA ATTTCTTCCTTGCTGGC-3'	This study, to generate $ERCC1_{pSIM1}$	N/A
ERCC1 pSIM2 FWD: 5'- CCGGGGCAAAATCCAACAGCGCCGCTGCGAGC CCTCGGCAGAGGGGCA-3'	This study, to generate $ERCC1_{pSIM2}$	N/A
ERCC1 pSIM2 REV: 5'- TGCCCCTCTGCCGAGGGCTCGCAGCGGCGCTGT TGGATTTTGCCCCGG-3'	This study, to generate $ERCC1_{pSIM2}$	N/A
ERCC1 EcoRI 5': 5'- taagcagaattcATGGACCCTGGGAAGGACAAAG- 3'	This study, to clone ERCC1 into pDONR207-3X-ΔN11-3X-SUMO2-ΔGG-CENPW	N/A
ERCC1 STOP NotI 3': 5'- tgcttagcggccgcTCAGGGTACTTTCAAGAAGGG- 3'	This study, to clone ERCC1 into pDONR207-3X-ΔN11-3X-SUMO2-ΔGG-CENPW	N/A
**Recombinant DNA**		
pSpCas9(BB)-2A-GFP (PX458)	Addgene	48138
pDONR223-ERCC1 ORF	Sigma-Aldrich MISSION® TRC3 Human ORF Collection	Legacy clone name: ORF001395.1_s300c1
pU6-gRNA/PGK-Puro-2A-BFP with gRNA sequence: 5'- ATCCAAGAAAGCCAGCCCAGAGG-3'	Sigma-Aldrich Human Sanger Arrayed Whole Genome Lentiviral CRISPR library	Clone ID: 226656931
pBABE-puro-$ERCC1_{WT}$-GFP	This study	N/A
pBABE-puro-$ERCC1_{pSIM1}$-GFP	This study	N/A
pBABE-puro-$ERCC1_{pSIM2}$-GFP	This study	N/A
pBABE-puro-$ERCC1_{pSIM1+2}$-GFP	This study	N/A
pBABE-puro-GFP-3xSUMO2-$ERCC1_{WT}$	This study	N/A
pBABE-puro-GFP-3xSUMO2-$ERCC1_{SIM}$	This study	N/A
pCW57.1-GFP-$SENP6_{WT}$-kd-resistant	[3]	N/A
pCW57.1-GFP-$SENP6_{CD}$-kd-resistant	[3]	N/A
pHis-TEV30a-His10-ΔN11-SUMO2-trimer	[4]	N/A
pDONR207-3X-ΔN11-3X-SUMO2-ΔGG-CENPW	This study	N/A

[a]IB: immunoblotting; [b]IF: immunofluorescence

References

1 Vertegaal, A. C. O. *et al.* A Proteomic Study of SUMO-2 Target Proteins. *J of Biol Chem* **279**, 33791-33798 (2004).

2 Vyas, R. *et al.* RNF4 is required for DNA double-strand break repair in vivo. *Cell Death & Differentiation* **20**, 490-502 (2013).

3 Liebelt, F. *et al.* The poly-SUMO2/3 protease SENP6 enables assembly of the constitutive centromere-associated network by group deSUMOylation. *Nat Commun* **10**, 3987 (2019).

4 Eifler, K. *et al.* SUMO targets the APC/C to regulate transition from metaphase to anaphase. *Nat Commun* **9**, 1119 (2018).
