# Supplementary material for: SENP6 regulates localization and nuclear condensation of DNA damage response proteins by group deSUMOylation
Source: Nat Commun. 2023 Sep 22;14:5893. doi: 10.1038/s41467-023-41623-w (PMC10514054; doi:10.1038/s41467-023-41623-w)
Supplement: Supplementary file 3 — Reporting Summary [file 41467_2023_41623_MOESM3_ESM.pdf]

nature portfolio

Corresponding author(s): Alfred Vertegaal

Last updated by author(s): Sep 7, 2023

Reporting Summary

Nature Portfolio wishes to improve the reproducibility of the work that we publish. This form provides structure for consistency and transparency in reporting. For further information on Nature Portfolio policies, see our Editorial Policies and the Editorial Policy Checklist.

Statistics

For all statistical analyses, confirm that the following items are present in the figure legend, table legend, main text, or Methods section.

n/a	Confirmed	
☐	☒	The exact sample size (n) for each experimental group/condition, given as a discrete number and unit of measurement
☐	☒	A statement on whether measurements were taken from distinct samples or whether the same sample was measured repeatedly
☐	☒	The statistical test(s) used AND whether they are one- or two-sided<br>*Only common tests should be described solely by name; describe more complex techniques in the Methods section.*
☒	☐	A description of all covariates tested
☐	☒	A description of any assumptions or corrections, such as tests of normality and adjustment for multiple comparisons
☐	☒	A full description of the statistical parameters including central tendency (e.g. means) or other basic estimates (e.g. regression coefficient) AND variation (e.g. standard deviation) or associated estimates of uncertainty (e.g. confidence intervals)
☐	☒	For null hypothesis testing, the test statistic (e.g. F, t, r) with confidence intervals, effect sizes, degrees of freedom and P value noted<br>*Give P values as exact values whenever suitable.*
☒	☐	For Bayesian analysis, information on the choice of priors and Markov chain Monte Carlo settings
☒	☐	For hierarchical and complex designs, identification of the appropriate level for tests and full reporting of outcomes
☐	☒	Estimates of effect sizes (e.g. Cohen's d, Pearson's r), indicating how they were calculated

Our web collection on statistics for biologists contains articles on many of the points above.

Software and code

Policy information about availability of computer code

Data collection	Leica Application Suite X www.leica-microsystems.com
Data analysis	Graphpad Prism9 www.graphpad.com<br>ImageJ2 (v1.53f51) from Fiji www.fiji.sc<br>Cytoscape 3.8.0 www.cytoscape.org

For manuscripts utilizing custom algorithms or software that are central to the research but not yet described in published literature, software must be made available to editors and reviewers. We strongly encourage code deposition in a community repository (e.g. GitHub). See the Nature Portfolio guidelines for submitting code & software for further information.

Data

Policy information about availability of data

All manuscripts must include a data availability statement. This statement should provide the following information, where applicable:

- Accession codes, unique identifiers, or web links for publicly available datasets
- A description of any restrictions on data availability
- For clinical datasets or third party data, please ensure that the statement adheres to our policy

Data availability statement provided in manuscript: Data are available in the Source Data file or from the corresponding author upon request.

Research involving human participants, their data, or biological material

Policy information about studies with human participants or human data. See also policy information about sex, gender (identity/presentation), and sexual orientation and race, ethnicity and racism.

Reporting on sex and gender	Not applicable
Reporting on race, ethnicity, or other socially relevant groupings	Not applicable
Population characteristics	Not applicable
Recruitment	Not applicable
Ethics oversight	Not applicable

Note that full information on the approval of the study protocol must also be provided in the manuscript.

Field-specific reporting

Please select the one below that is the best fit for your research. If you are not sure, read the appropriate sections before making your selection.

[x] Life sciences [ ] Behavioural & social sciences [ ] Ecological, evolutionary & environmental sciences

For a reference copy of the document with all sections, see nature.com/documents/nr-reporting-summary-flat.pdf

Life sciences study design

All studies must disclose on these points even when the disclosure is negative.

Sample size	Sample sizes were chosen according to common practice in the field.
Data exclusions	No data were excluded from the analyses.
Replication	Experiments were independently repeated, yielding reproducible data. The exact sample sizes (n) are specified in the legends.
Randomization	Not applicable
Blinding	Not applicable

Reporting for specific materials, systems and methods

We require information from authors about some types of materials, experimental systems and methods used in many studies. Here, indicate whether each material, system or method listed is relevant to your study. If you are not sure if a list item applies to your research, read the appropriate section before selecting a response.

Materials & experimental systems

n/a	Involved in the study	
[ ]	[x]	Antibodies
[ ]	[x]	Eukaryotic cell lines
[x]	[ ]	Palaeontology and archaeology
[x]	[ ]	Animals and other organisms
[x]	[ ]	Clinical data
[x]	[ ]	Dual use research of concern
[x]	[ ]	Plants

Methods

n/a	Involved in the study	
[x]	[ ]	ChIP-seq
[x]	[ ]	Flow cytometry
[x]	[ ]	MRI-based neuroimaging

Antibodies

Antibodies used

anti-SENP6 (IBa 1:100; IFb 1:50) Sigma-Aldrich WHP0026054M1
anti-SENP7 (IB 1:1000) Bethyl A302-995A
anti-SUMO2/3 8A2 (IB 1:250) University of Iowa 8A2
anti-SUMO2/3 2277 (IF 1:100) Eurogentec Vertegaal et al. 2004 J Biol Chem 279(32):33791-8

anti-SUMO1 2C7 (IB 1:1000; IF 1:100) Zymed laboratories Inc. 18-2306
anti-PML (IB 1:100; IF 1:500) Bethyl A301-167A

anti-PML (IB 1:1000; IF 1:1000) MBL M041-3
anti-γH2AX (IB 1:500; IF 1:500) Merck Millipore 05-636
anti-γH2AX (IB: 1:500) Cell signaling technology 9718
anti-RNF4 (IB 1:3000) Eurogentec Vyas et al. Cell Death Diff 2013;20(3):490-502.
anti-ERCC1 (IF 1:100) Abcam ab129267
anti-ERCC1 (IB 1:500-1:1000) Cell signaling technology 12345
anti-BLM (IB 1:1000; IF 1:200) Abcam ab2179
anti-XPF (IB 1:500) Thermo Fisher Scientific MS-1381
anti-XPF (IF 1:100) Santa Cruz sc-136153
anti-MUS81 (IB 1:500-1:1000; IF 1:250) ImmuQuest IQ285
anti-53BP1 (IB 1:1000; IF 1:500) Bethyl A300-272A
anti-BRCA1 (IB: 1:500) Cell signaling technology 9010
anti-BRCA1 (IF 1:1000) Merck Millipore 07-434
anti-RAP80 (IB 1:1000; IF 1:200) Bethyl A300-763A
anti-MDC1 (IB 1:1000; IF 1:500) Bethyl A300-052A
anti-CtIP (IB 1:1000; IF 1:200) Bethyl A300-488A
anti-EME1 (IB 1:1000) ImmuQuest IQ284
anti-BARD1 (IB 1:1000) Bethyl A300-263A
anti-Sp100 (IB 1:1000) Chemicon international AB1380
anti-RAD51 (IB 1:500) Cell signaling technology 8875
anti-Ubiquitinated proteins FK2 (IB 1:1000) Merck Millipore 04-263
anti-CENP-C (IF 1:100) Merck Millipore MABE1114
anti-GFP (IB 1:5000; IF 1:500) Novus Biololgicals NB600-308
anti-Cas9 (IB 1:1000) Cell Signaling technology 14697
Alexa-Fluor 568 goat-anti-rabbit / goat-anti-mouse (IF 1:250) Invitrogen A11004 / A11011
Alexa-Fluor 488 goat-anti-rabbit / goat-anti-mouse (IF 1:250) Invitrogen A11034 / A11001

Validation: The antibodies described above are validated on the websites of the manufacturer and in the cited articles.

Eukaryotic cell lines

Policy information about cell lines and Sex and Gender in Research

Cell line source(s)	U2OS, 293T and 293GP were derived from the ATCC
Authentication	Cell lines have been authenticated via STR profiling using 10 different markers
Mycoplasma contamination	Cell lines have been tested to be free of mycoplasma
Commonly misidentified lines (See ICLAC register)	Not applicable
